# Supplementary material for: Realization of topological Mott insulator in a twisted bilayer graphene lattice model
Source: Nat Commun. 2021 Sep 16;12:5480. doi: 10.1038/s41467-021-25438-1 (PMC8446059; doi:10.1038/s41467-021-25438-1)
Supplement: Supplementary file 1 — Supplementary Information [file 41467_2021_25438_MOESM1_ESM.pdf]

Supplementary Information for:
Realization of Topological Mott Insulator in a Twisted Bilayer Graphene Lattice Model

Chen *et al.*

Supplementary Note 1: DMRG Results

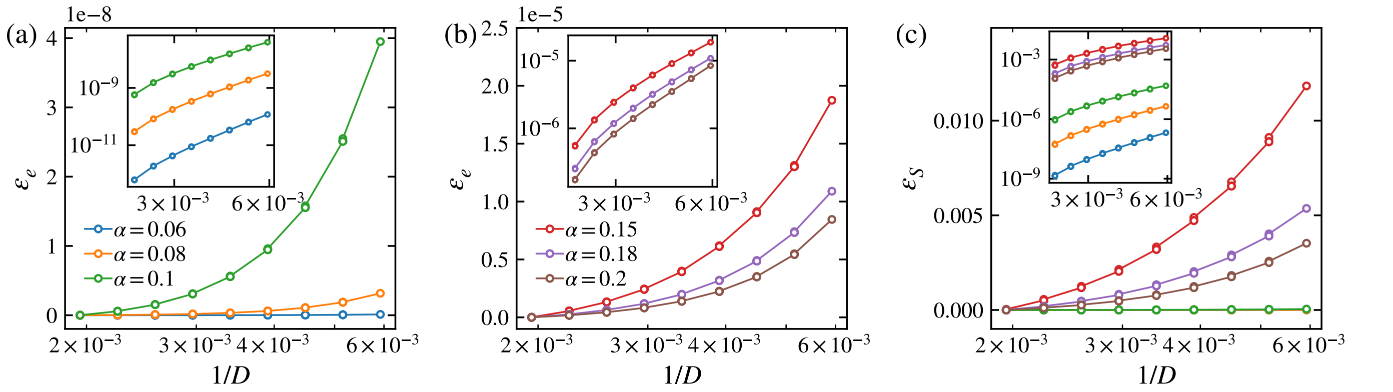

Supplementary Figure 1. **DMRG convergence check.** (a) Energy differences between the intermediate sweep I and the final sweep I_f, $\epsilon_e = e(I) - e(I_f)$ as a function of $1/D$ in the stripe phase. The inset shows ϵ_e in a logarithmic scale. (b) Same layout as panel (a) but in the QAH phase. (c) Entanglement entropy differences between the intermediate sweep I and the final sweep I_f, $\epsilon_S = S_E(I) - S_E(I_f)$ as a function of $1/D$ in the stripe phase. The inset shows ϵ_S in a logarithmic scale.

In this section, we present more DMRG results further supporting the conclusion in the main text.

DMRG data convergence. Firstly, we show in Supplementary Figure 1 the computed energy and entanglement entropy vs. bond dimension $1/D$. in our DMRG calculations. In practice, to ensure convergence of the data, we ramp up the bond dimensions D in the course of optimization, i.e., $D(I) = D(0) \cdot a^I$, where a is a parameter controlling the increasement of $D(I)$ in the I-th step, with $I \in 0, 1, \cdots, I_f$. To be specific, starting with an initial bond dimension $D(0)$, we increase the bond dimension $D(I)$, step by step, until the final bond dimension $D(I_f)$ is reached. In practical calculations, we set $D(0) = 128$, $a = 2^{1/5}$, and $D(I_f) = 512(1024)$ for width 4(6) cylinder, the results are very well converged. In addition, at each intermediate step I, we perform 5 sweeps before moving to the next step $I + 1$ with increased bond dimension $D(I + 1)$.

In Supplementary Figure 1(a,b), we show the differences of the calculated ground-state energy $\epsilon_e = e(I) - e(I_f)$ versus the retained bond dimension $1/D$, from which one can observe that, for both stripe [panel(a)] and QAH [panel (b)] phases, the energy have well-converged within $\epsilon_e \sim 10^{-12\sim-9}$ and $10^{-7\sim-6}$, respectively. The entanglement entropy differences ΔS_E are shown in Supplementary Figure 1(c), which also show good convergence, with $\epsilon_S \sim 10^{-9\sim-3}$, depending on the specific α parameters.

Single-particle Green's function. In Supplementary Figure 2 we show the single-particle Green's function results on YC4 geometry, where $G_{\lambda,\lambda'}(x\mathbf{L_2}) = \langle c^\dagger_{\mathbf{R}+\delta_\lambda} c_{\mathbf{R}+x\mathbf{L_2}+\delta_{\lambda'}} \rangle$ is computed by DMRG, with $\mathbf{R} = \frac{W}{2}(\mathbf{2L_1} - \mathbf{L_2})$. From Supplementary Figure 2, we find a very clear exponential decay of all four elements of the 2×2 Green's function matrix, with a rather short correlation length $\xi < 1$ (in the unit of NN edge of honeycomb lattice). These results are consistent with the flat entanglement entropy shown in the inset of Fig. 2 (b) in main text, pointing to a ground state with nonzero charge gap in the bulk.

QAH state with Chern number $C = -1$. Since the QAH state in the large-α phase spontaneously breaks the time-reversal symmetry, the topological states can thus have Chern numbers $C = 1$ and -1. In Supplementary Figure 3 we show the ground state with $C = -1$, which have roughly 50% probability to appear in our calculations. From Supplementary Figure 3(a), we find the directional circular currents, signaling the TRS breaking, follow rightly the opposite chirality to the QAH state shown in Fig. 1(e) of the main text where the $C = 1$ QAH state is realized. Another distinction is the fractional charge zero modes on the edges of cylinders, upon flux insertion. For the $C = -1$ state, when we thread a flux from left to right [following exactly the inset of Fig. 3(a) in main text], we find an unit charge $\Delta Q = 1$ is pumped from right edge to the left. This is revealed explicitly in Supplementary Figure 3(b), where we find the end charge increases from $1/2$ to $3/2$ on the left edge, while decreases from $-1/2$ to $-3/2$ on the right edge.

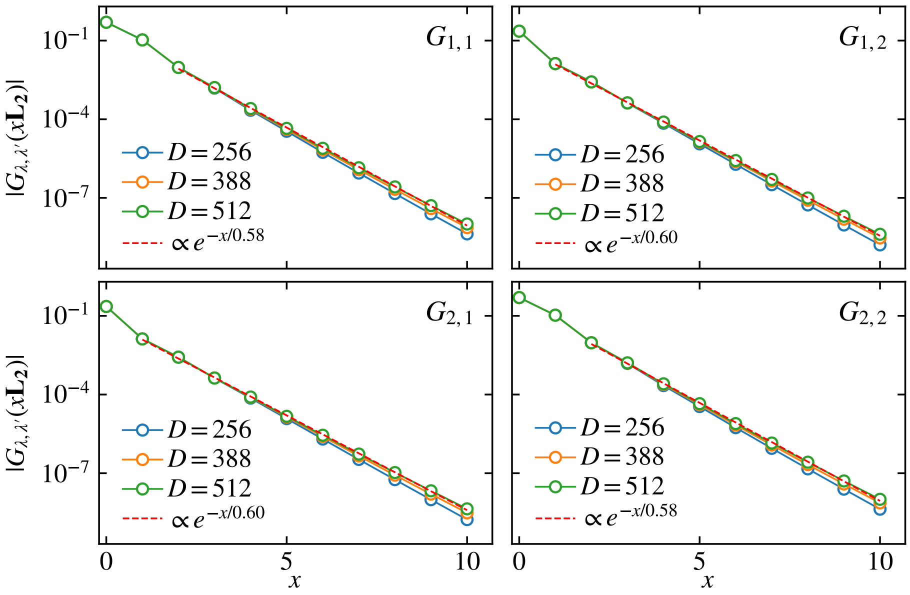

Supplementary Figure 2. **Single-particle Green's function.** In a YC4 $\times$ 24 $\times$ 2 system with $\alpha = 0.15$ (QAH phase), single-particle Green's functions $G_{\lambda,\lambda'}(x\mathbf{L_2})$ are calculated, which are shown to be well converged vs. D. The four components $G_{1,1}, G_{1,2}, G_{2,1}, G_{2,2}$ are plotted versus x in (a-d) panels, which all decay exponentially as $\propto e^{-x/\xi}$, with similar correlation lengths $\xi \simeq 0.6$.

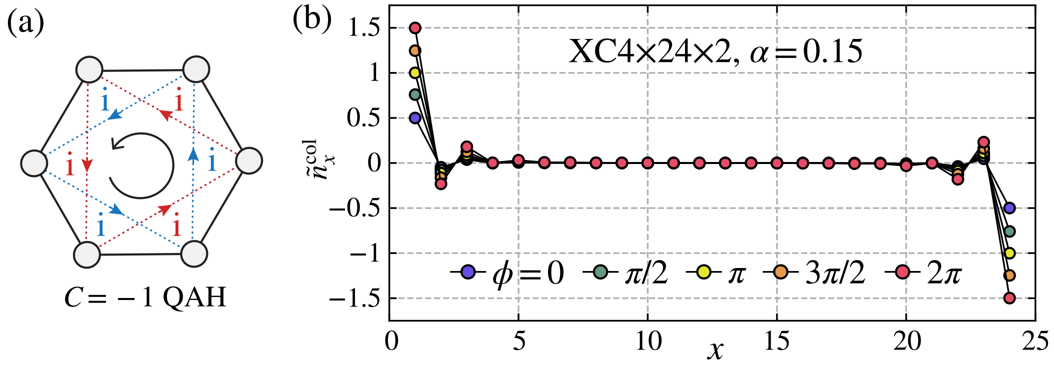

Supplementary Figure 3. **Real-space charge distributions.** (a) The rotation of loop current is opposite with the one ($C = 1$ QAH) in main text [c.f. inset of Fig. 1(e)], thus a $C = -1$ QAH state. (b) In a XC4 $\times$ 24 $\times$ 2 system with $\alpha = 0.15$ (QAH phase), the charge distributions for different ϕ-flux threading the cylinder hole. An electron is pumped from the right edge to the left, resulting $\pm\frac{3}{2}$ charges in both end, corresponding to a $C = -1$ QAH state.

Supplementary Note 2: Stripe phase with small α

In this section, we study the possible ground state of the Hamiltonian Eq. (1) in main text with small α. Here, we follow the analysis in Ref. [1]. The Hamiltonian can be expanded as

$$H = H_0 + 2\alpha H_1 + \alpha^2 H_2 \tag{1}$$

$$H_0 = \sum_{\hexagon} (Q_{\hexagon} - 1)^2 \tag{2}$$

$$H_1 = \sum_{\hexagon} T_{\hexagon}(Q_{\hexagon} - 1) \tag{3}$$

$$H_2 = \sum_{\hexagon} (T_{\hexagon})^2 \tag{4}$$

Note that $T_{\hexagon}$ commutes with $Q_{\hexagon}$ because the hopping of $T_{\hexagon}$ occurs inside the hexagon, and thus does not change the total charges on the six vertices of the hexagon. Therefore, $T_{\hexagon}(Q_{\hexagon}-1)=(Q_{\hexagon}-1)T_{\hexagon}$.

When α is small, we can treat H_1 and H_2 as perturbations with respect to the leading term H_0. At the half filling, the ground state of H_0 is given by the constraints $(Q_{\hexagon}-1)|\Psi_0\rangle=0$ for all the hexagons $\hexagon$ and thus the corresponding energy $E_0=0$. This constraint is satisfied by a large manifold of states, including both the stripe and sublattice polarized states. Now, consider the perturbation of H_1 and H_2 within the manifold of the degenerate states. For any state Ψ_0 in this manifold, $(Q_{\hexagon}-1)|\Psi_0\rangle=0$. As a consequence, both the first and second order perturbations of H_1 vanishes:

$$\sum_{\hexagon}\langle\Psi_0'|T_{\hexagon}(Q_{\hexagon}-1)|\Psi_0\rangle=0 \tag{5}$$

$$\sum_{\hexagon}\sum_{\hexagon'}\sum_{n\notin\Psi_0}\frac{1}{E_0-E_n}\times\langle\Psi_0'|(Q_{\hexagon}-1)T_{\hexagon}|n\rangle\langle n|T_{\hexagon'}(Q_{\hexagon'}-1)|\Psi_0\rangle=0 \tag{6}$$

where Ψ_0 and Ψ_0' are two arbitrary orthogonal states inside the ground state manifold of H_0, and $|n\rangle$ labels the excited state of H_0. Up to $O(\alpha^2)$, we also need to include the first order correction of H_2, i.e., $\sum_{\hexagon}\langle\Psi_0'|\left(T_{\hexagon}\right)^2|\Psi_0\rangle$. After expanding the square form into the four-fermion terms, it is obvious that only the following terms

$$\sum_{\mathbf{R}}\sum_{l=1}^{6}\sum_{\eta=\pm1}\sum_{\lambda}c^\dagger_{\mathbf{R}+\delta_l,\lambda}c_{\mathbf{R}+\delta_{l+\eta},\lambda}c^\dagger_{\mathbf{R}+\delta_{l+\eta},\lambda}c_{\mathbf{R}+\delta_l,\lambda}$$

survive, where the index $\mathbf{R}$ refers to the hexagon and $\mathbf{R}+\delta_l$ and $\mathbf{R}+\delta_{l\pm1}$ are two neighboring vertices of this hexagon. These terms do not change the total charge of each hexagon. It is obvious that their first order correction is 1 if the site $\mathbf{R}+\delta_l$ is occupied and the site $\mathbf{R}+\delta_{l\pm1}$ is empty, and becomes 0 otherwise. Therefore, among the states in which $Q_{\hexagon}=1$ for every hexagon, this correction is minimized by decreasing number of "dangling" bonds that connect an occupied site and an empty one. At the half filling, the number of such bonds becomes smallest for the stripe phase, as shown in Fig. 1(f) of the main text. The corresponding first order energy correction per site is thus

$$\delta E/N=\alpha^2U_0, \tag{7}$$

and plotted as the blue dashed curve in Fig. 2(a) of the main text.

Supplementary Note 3: Mean Field Approximation with Large α

When α becomes larger, the perturbation theory in the previous section fails. Our DMRG calculation has revealed that the QAH state appears with $\alpha\gtrsim0.12$ and furthermore, the state can be approximated as the Slater determinant of the Bloch states. Motivated by these DMRG results, we consider a C_3 symmetric tight binding model with the hopping constants up to the *fifth* nearest neighbor. As shown in Supplementary Figure 4, the hopping terms are

$$\begin{aligned}
H_t=&\,H_1+H_2+H_3+H_4+H_5 \qquad (8)\\
H_1=&\sum_{\mathbf{R}}t_1c^\dagger_{\mathbf{R}+\delta_2}\left(c_{\mathbf{R}+\delta_1}+c_{\mathbf{R}+\mathbf{L}_1+\delta_1}+c_{\mathbf{R}+\mathbf{L}_1-\mathbf{L}_2+\delta_1}\right)+h.c.\\
H_2=&\sum_{\mathbf{R}}t_2c^\dagger_{\mathbf{R}+\delta_2}\left(c_{\mathbf{R}+\mathbf{L}_1-\mathbf{L}_2+\delta_2}+c_{\mathbf{R}+\mathbf{L}_2+\delta_2}+c_{\mathbf{R}-\mathbf{L}_1+\delta_2}\right)+h.c.\\
&+t_2'c^\dagger_{\mathbf{R}+\delta_1}\left(c_{\mathbf{R}+\mathbf{L}_2-\mathbf{L}_1,1}+c_{\mathbf{R}-\mathbf{L}_2+\delta_1}+c_{\mathbf{R}+\mathbf{L}_1+\delta_1}\right)+h.c.\\
H_3=&\sum_{\mathbf{R}}t_3c^\dagger_{\mathbf{R}+\delta_2}\left(c_{\mathbf{R}+2\mathbf{L}_1-\mathbf{L}_2+\delta_1}+c_{\mathbf{R}+\mathbf{L}_2+\delta_1}+c_{\mathbf{R}-\mathbf{L}_2+\delta_1}\right)+h.c.\\
H_4=&\sum_{\mathbf{R}}t_4c^\dagger_{\mathbf{R}+\boldsymbol{\delta}_2}\left(c_{\mathbf{R}+\mathbf{L}_1+\mathbf{L}_2+\delta_1}+c_{\mathbf{R}+2\mathbf{L}_1-2\mathbf{L}_2+\delta_1}+c_{\mathbf{R}-\mathbf{L}_1+\delta_1}\right)+h.c.\\
&+t_4'c^\dagger_{\mathbf{R}+\delta_2}\left(c_{\mathbf{i}+2\mathbf{L}_1,\mathbf{1}}+c_{\mathbf{i}+\mathbf{L}_1-2\mathbf{L}_2,\mathbf{1}}+c_{\mathbf{i}+\mathbf{L}_2-\mathbf{L}_1,\mathbf{1}}\right)+h.c.\\
H_5=&\sum_{i}t_5c^\dagger_{\mathbf{i},\mathbf{2}}\left(c_{\mathbf{i}+\mathbf{L}_1+\mathbf{L}_2,\mathbf{2}}+c_{\mathbf{i}+\mathbf{L}_1-2\mathbf{L}_2,\mathbf{2}}+c_{\mathbf{i}+\mathbf{L}_2-2\mathbf{L}_1,\mathbf{2}}\right)+h.c.\\
&+t_5'c^\dagger_{\mathbf{i},\mathbf{1}}\left(c_{\mathbf{i}+2\mathbf{L}_1-\mathbf{L}_2,\mathbf{1}}+c_{\mathbf{i}-\mathbf{L}_2-\mathbf{L}_1,\mathbf{1}}+c_{\mathbf{i}+2\mathbf{L}_2-\mathbf{L}_1,\mathbf{1}}\right)+h.c.
\end{aligned}$$

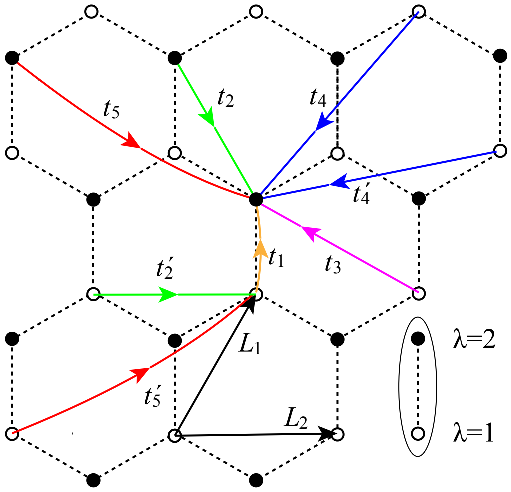

Supplementary Figure 4. **Hopping terms in the mean field theory.** Not all the hoppings are presented, as they can be obtained by the C_3 rotation symmetry applied on the sketched ones.

Supplementary Table 4. **Variational parameters and the Green's function**. For $\alpha = 0.15$ case, the variational parameters of hopping amplitudes and the Green's function obtained from both mean-field and DMRG calculations are listed up to 3rd nearest neighbor.

	varitional parameters	Green's function (mean-field)	Green's function (DMRG)
NN	$t_1/U_0 \simeq 0.2i$	$0.232i$	$0.224i$
NNN	$t_2/U_0 \simeq 0.116i$	$0.106i$	$0.099i$
3rd NN	$t_3/U_0 \simeq -0.102i$	$-0.080i$	$-0.074i$

where H_l ($l = 1, \cdots, 5$) are the hopping terms between nearest neighbors, next nearest neighbors, ..., up to the 5th nearest neighbors, respectively. The index $\mathbf{R}$ labels the position of unit cell, and $\lambda = 1$ (or 2) is the index of the sublattices.

This tight binding model can be diagonalized in the momentum space, and the chemical potential μ is fixed by the particle number. The tight binding model produces the many-body ground state as

$$|\psi\rangle = \prod_{\substack{i,\mathbf{k} \\ E_i(\mathbf{k})<\mu}} d_i^\dagger(\mathbf{k})|\emptyset\rangle$$

where $i = 1$ or 2 labels the two bands of the tight binding model H_t. Applying the variational mean field approximation, we minimize $E_{\rm mf} = \langle\psi|H|\psi\rangle$ with respect to all the hopping parameters in H_t. Here, H is the interaction Hamiltonian in Eq. (1) of the main text.

For all the possible values of α, we numerically found out that all the hopping parameters are purely imaginary and $t_2 = t_2'$. Furthermore, the hoppings beyond the 3rd nearest neighbor are tiny, and thus can be neglected in H_t. We have found that $E_{\rm mf}$ is minimized only when the hoppings of H_t are imaginary and thus lead to the QAH state. Therefore, H_t is qualitatively similar to the Haldane model up to a $U(1)$ gauge transformation.

The expectation value $E_{\rm mf}$ is plotted as the red dashed curve in Fig. 1(a) of the main text. The relative difference between $E_{\rm mf}$ and the energy produced by DMRG becomes smaller as α increases. Our variational mean field calculations suggest that this difference originates from the quantum fluctuation, that also becomes smaller as α increases as suggested in Fig. 3(e) of the main text.

Besides the variational energy, we have also compared the single-particle Green's functions obtained from mean-field calculations and DMRG. As shown in Supplementary Table I, the hopping amplitudes (as variational parameters in the mean-field calculations) are listed, up to 3rd nearest neighbor in the first column. The corresponding single-particle Green's functions from both the mean-field and DMRG calculations show excellent agreement, with differences $< 0.01i$, confirming the effectiveness and accuracy of the mean-field theory in understanding the QAH phase in the interaction-only TBG superlattice model.

Supplementary Note 4: Half-charge Zero Modes on the Edge

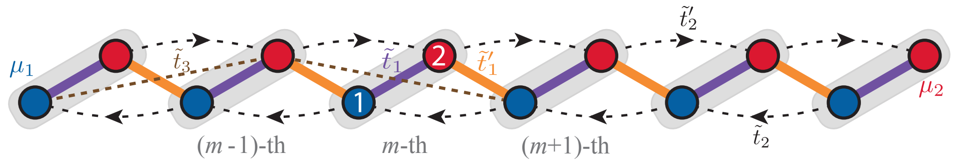

Supplementary Figure 5. **The generalized SSH model**. Illustration of the 1D model $H_{\rm 1D}(k)$, with both NN $(\tilde{t}_1, \tilde{t}'_1)$ and NNN $(\tilde{t}_2, \tilde{t}'_2)$ hopping amplitudes, as well as sublattice chemical potential μ_1 and μ_2.

In this section, we provide a detailed analysis of the particle occupation number and half-charge zero modes on the edges, based on our tight binding model on a cylinder. Here, we focus on the XC geometry with W unit cells along the periodic direction, so that

$$c_{\mathbf{R}+\delta_\lambda} \equiv c_{\mathbf{R}+W\mathbf{L}_2+\delta_\lambda}$$

where $\lambda = 1$ or 2 refers to the sublattice. For the sake of simplicity, in the following discussions, we only keep H_t [c.f. Supplementary Eq. (8)] up to the *third* order, denoted as $H_t^{(3)}$. Rewriting $H_t^{(3)}$ through Fourier transformation along $\mathbf{L}_2$ direction

$$c_{m,\lambda}(k) = \frac{1}{\sqrt{W}} \sum_{l=1}^{W} c_{l\mathbf{L}_1+m\mathbf{L}_2+\delta_\lambda} e^{-ikl} \ ,$$

we arrive at the Hamiltonian in the hybridized (m, k) space,

$$\begin{aligned} H_t^{(3)} = \sum_{m,k} \Big[& t_1(1+e^{ik}) c^\dagger_{m,2}(k) c_{m,1}(k) + t_1 e^{ik} c^\dagger_{m,2}(k) c_{m-1,1}(k) \\ & + t_2 e^{-ik} c^\dagger_{m,2}(k) c_{m,2}(k) + (t_2^* e^{-ik} + t_2)\, c^\dagger_{m,2}(k) c_{m+1,2}(k) \\ & + t_2 e^{ik} c^\dagger_{m,1}(k) c_{m,1}(k) + (t_2^* + t_2 e^{-ik})\, c^\dagger_{m,1}(k) c_{m+1,1}(k) \\ & + t_3(1+e^{2ik})\, c^\dagger_{m,2}(k) c_{m-1,1}(k) + t_3 c^\dagger_{m,2}(k) c_{m+1,1}(k) \Big] + h.c. \end{aligned} \tag{9}$$

We recognize $H_t^{(3)}$ as a summation of W decoupled 1D chains, i.e., $H_t^{(3)} = \sum_k H_{\rm 1D}(k)$ with

$$\begin{aligned} H_{\rm 1D}(k) = & \sum_m (\tilde{t}_1 c^\dagger_{m,2}(k) c_{m,1}(k) + \tilde{t}'_1 c^\dagger_{m,2}(k) c_{m+1,1}(k) + \tilde{t}_3 c^\dagger_{m,2}(k) c_{m-1,1}(k)) + h.c. \\ & + \sum_m (\tilde{t}_2\, c^\dagger_{m,1}(k) c_{m+1,1}(k) + \tilde{t}'_2\, c^\dagger_{m,2}(k) c_{m+1,2}(k)) + h.c. \\ & + \sum_{m,\lambda} \mu_\lambda c^\dagger_{m,\lambda} c_{m,\lambda} \ . \end{aligned} \tag{10}$$

This 1D generalized SSH Hamiltonian is illustrated in Supplementary Figure 5, where the hopping amplitudes are

$$\tilde{t}_1 = t_1(1+e^{ik}) \qquad \tilde{t}'_1 = t_3 \qquad \tilde{t}_3 = t_1 e^{ik} + t_3(1+e^{2ik}) \qquad \tilde{t}_2 = (t_2^* + t_2 e^{-ik}) \tag{11}$$

$$\tilde{t}'_2 = (t_2 + t_2^* e^{-ik}) \qquad \tilde{\mu}_1 = 2{\rm Re}(t_2 e^{ik}) \qquad \mu_2 = 2{\rm Re}(t_2 e^{-ik}). \tag{12}$$

Zero modes on the chiral edge at π momentum. In Supplementary Figure 6(a,b), we show the dispersion of $H_t^{(2)}$ [Supplementary Eq. (9)] on an open-ended cylinder as a function of k, the momentum along the periodic direction. While the bulk states are clearly gapped, there exists two branches of chiral edge modes that are gapless [c.f., insets of Supplementary Figure 6(a) for their charge distributions localized on the edge], corresponding to the bulk QAH topological state. Interestingly, the two edge modes are found to be degenerate only at $k = \pi$. where the two branches cross. This can be understood as follows, by introducing a sublattice dependent gauge transformation τ_z, under which $c_{\mathbf{R}+\delta_l} \longrightarrow (-)^l c_{\mathbf{R}+\delta_l}$. It is obvious that the interaction H [Eq. (1) of the main text] is invariant under the combined symmetry $\tau_z C_{2z}$, where C_{2z} is the two-fold rotation around the center of a plaquette. Furthermore, the mean field Hamiltonian $H_t^{(3)}$ with all imaginary hoppings is also invariant under $\tau_z C_{2z}$. As a consequence, the two edge modes must cross at C_{2z} invariant momentum, i.e., at $k = \pi$. This crossing also sets the

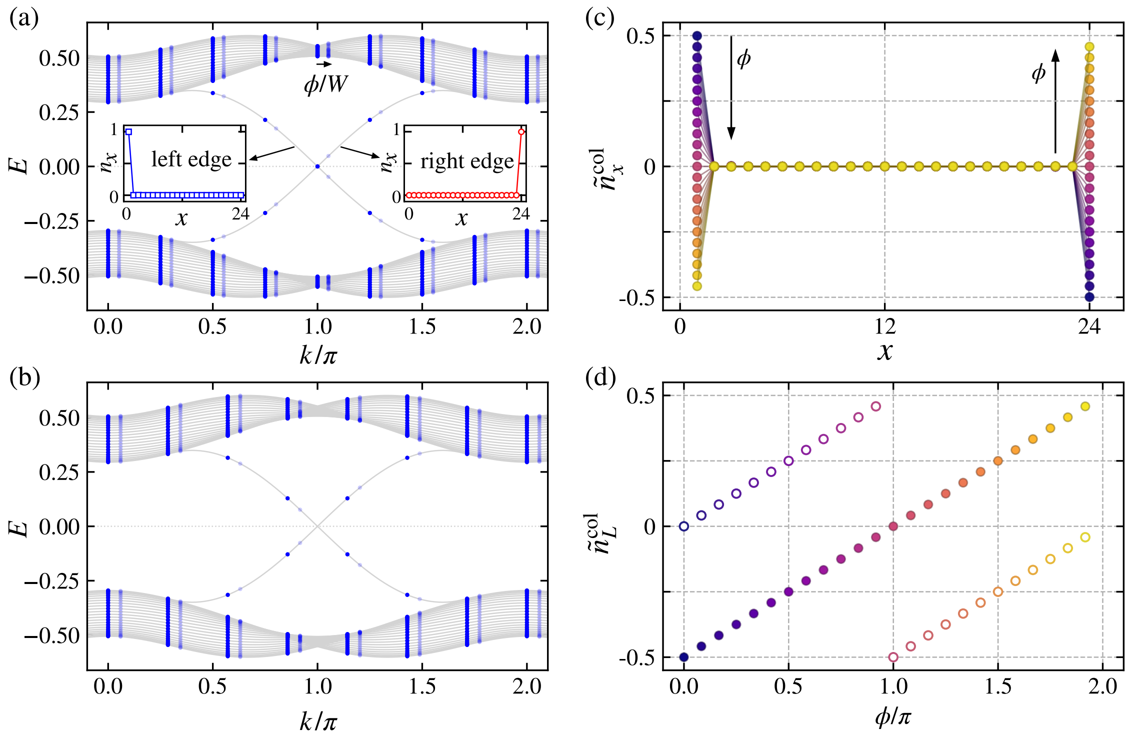

Supplementary Figure 6. **Flux insertion in the tight-binding model.** (a) The spectrum of $H_t^{(3)}$ with even width $W = 8$ and hopping parameters $t_1 = 0.2\mathrm{i}$, $t_2 = 0.116\mathrm{i}$ and $t_3 = -0.102\mathrm{i}$, taken from the $\alpha = 0.15$ DMRG calculations. All the allowed momenta are colored dark blue, which are shifted to the light-blue dots by $\Delta k = \phi/W$ in the flux insertion case. The insets show the single-particle charge distributions of the two edge modes. (b) Same layout as panel (a) with odd width $W = 7$ otherwise. (c) For even width system ($W = 8$ here), the charge distribution over column index i is shown for flux $\phi \in [0, 2\pi)$, with the color code indicated by panel (d). (d) Number of charge at the right edge $\tilde{n}^{\mathrm{col}}_{i=L}$ for $W = 8$(filled) and $W = 7$(hollow) system.

chemical potential at the half filling if it is inside the insulating gap. For even W, the system has to occupy one of the two states at the crossing, so that $|n_1^{col} - n_L^{col}| = 1$, leading to the appearance of half charges on both sides. Notably, the conclusion of half-charge zero modes at $k = \pi$ holds only for even W since the edge state with $k = \pi$ does not exist if W is odd.

Flux insertion in the tight-binding model. Below, we stick to the more simpler $H_t^{(3)}$ and analyze the charge pumping therein through flux insertion. When W, the number of unit cell along the periodic direction, is finite, the set of all possible momenta is finite, with $k = \frac{2\pi n}{W}$ and $n = 0, 1, \cdots, W-1$ if the magnetic flux is absent [see the dark blue dots in Supplementary Figure 6(a,b)]. If W is odd, k can never be π, and thus the system only fills all the states below $E = 0$ in Supplementary Figure 6(b). Therefore, the two edges have the same number of particles. The situation is quite different for even W [c.f. Supplementary Figure 6(a)], where the system can only fill one of the two zero modes on each edges. Therefore, there is one more particle on one edge than the other, leading to the appearance of half-charge zero modes on both edges.

Furthermore, when flux ϕ is inserted, the momentum k shifts by ϕ/W [see the light-blue dots in Supplementary Figure 6(a,b)]. As shown in Supplementary Figure 6(c), for the even W case, as the flux increases in a 2π period, the half charge gradually fades away and disappears for $\phi = \pi$, which then reappear with its sign reversed. The net charge transfer ΔQ from the left to the right edge in the course of charge pumping is shown in Supplementary Figure 6(d).

On the other hand, for cylinders with odd W, the system starts from the initial state with no half-charge zero modes (as $k = \pi$ can not be selected due to the cylinder geometry), and the pumped charge undergoes a jump right at $\phi = \pi$, when the half-charge zero modes restore. As ϕ exceeds π, the half-charge zero mode switches to the left end (instead of the right one), and thus the $\tilde{n}_L^{\mathrm{col}}$ jumps from $+0.5$ to -0.5, and then gradually increases as the flux ϕ increases and vanishes again for $\phi = 2\pi$. We note that the "jump" of charge distribution in the flux insertion procedure happens in the tight-binding model $H_t^{(3)}$ [Supplementary Figure 6(d)] does not necessarily take place in the adiabatic DMRG simulations of XCW with W odd. As there we feed the ground state of previous flux ϕ as the initial state of next flux ϕ, and the charge pumping from one end to the other can be realized, without such abrupt "jump", in practice.

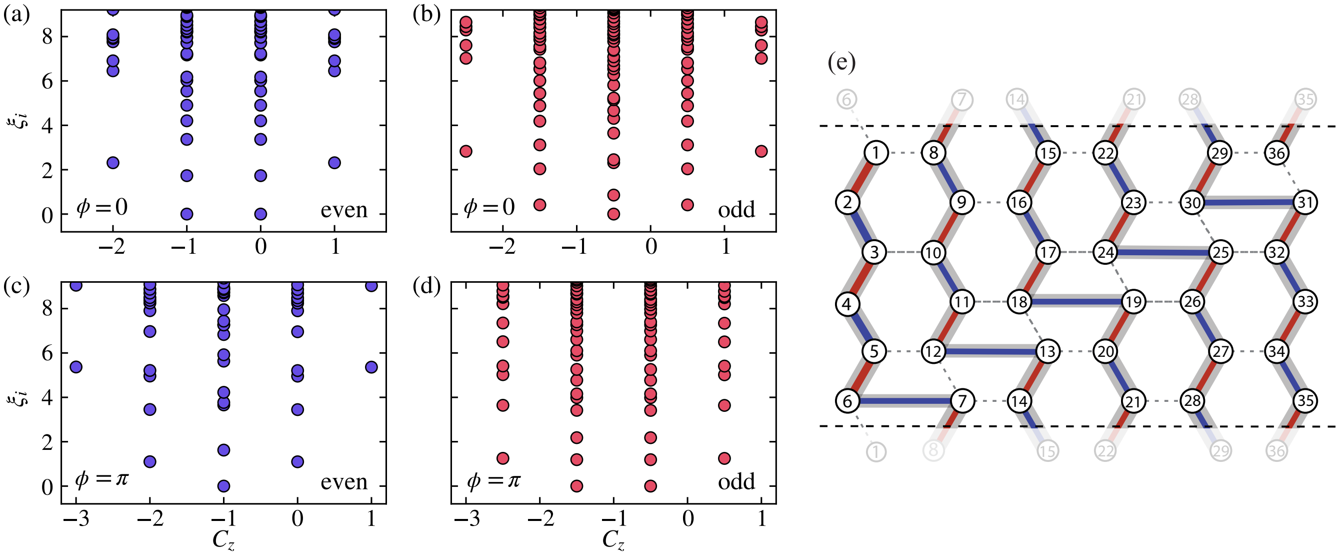

Supplementary Figure 7. **Degeneracies in the entanglement spectra.** In XC4 $\times$ 24 $\times$ 2 system with $\alpha = 0.15$ and $\phi = 0$, the entanglement spectra are shown when cut at an (a) even and (b) odd bond in the central regime of the DMRG snake path. (c,d) Same layout as (a,b) otherwise for the $\phi = \pi$ case. (e) The snake path of an XC3 $\times$ 6 $\times$ 2 cylinder is shown with site ordering explicitly labeled, and the even/odd bonds are colored with blue/red. The two horizontal dashed lines indicates the periodic boundary conditions along the y (vertical) direction of the cylinder geometry.

Symmetry protected half-charge zero modes and degeneracies of entanglement spectrum. This half-charge zero modes on both cylinder edges, which disappear at the inserted flux $\phi = \pi$, is also reflected in the bulk property, in terms of the entanglement spectrum degeneracy. As shown in Supplementary Figure 7(a,b), in a XC4 $\times$ 24 $\times$ 2 cylinder with $\alpha = 0.15$ (QAH phase), the entanglement spectrum on an even bond [c.f. panel (a)] exhibits a two-fold degeneracy, while that on the odd bond is non-degenerate. As one threads a flux through the cylinder, the two-fold degeneracy is lifted. However, and very interestingly, at $\phi = \pi$ the two-fold degeneracy reappears on the odd bond (with even one non-degenerate). This is in accordance with the absence of zero edge modes at $\phi = \pi$, as shown in Supplementary Figure 6(b).

[1] J. Kang, O. Vafek, *Phys. Rev. Lett.* **122**, 246401 (2019).
